# The Pathology of Fever—Reconcilement of the Nervous and Humoral Theories

**Published:** 1845-11-01

**Authors:** 


*October*, 1845.

### The Pathology of Fever—Reconcilement of the Nervous and Humoral Theories.

By the Editor.

Pathologists are divided as respects the pathology of Fever by two doctrines. The one doctrine regards the febrile state as involving, primarily and essentially, the nervous system. The other doctrine attributes the original and essential morbid condition to a perversion of the fluids. May it not be, that here, as in some other instances, these apparently opposing doctrines are capable of being reconciled, so that the claims of either will be satisfied?

It would be fruitless to inquire into the nature of the changes which constitute the proximate cause of Fever. In the present state of our knowledge this subject is involved in entire obscurity. They who advocate the nervous, and they who contend for the humoral doctrine, generally, alike forbear the attempt to designate the intimate morbid perversions, or modifications, in which the essential nature of the disease is supposed to consist. They only respectively insist, on the one side, that the disease is to be regarded as radically, or primarily involving the nervous system, and, on the other side, the fluids. Now may it not be true that both these are invariably concerned in producing the febrile state, and that either may take precedence in the catenation of changes?

For example, fever ensues from animal poison, as in jail or camp (Typhus) fever, or from the imbibition of marsh miasmata, as in remittents and intermittents, preceded by a period of latency or incubation. In these instances it seems most rational to think that the fluids are *first* affected.—But nervous disorder always characterizes the commencement of the febrile state. That is, the perversion of fluids manifests itself, first, by certain modifications of the nervous system—the latter, however, succeeding to the former. But although the former has precedence, the latis necessarily involved in the production and continuance of the febrile career. The nervous disorder holds an invariable position of intervention between the primary action of the morbid cause on the fluids, and the phenomena which characterize the febrile movement. Chills and rigors, general malaise, cephalalgia, lassitude, pain in the loins and extremities, nausea, &c., are all due directly to the nervous system. It is also highly probable that the interruption and perversion of the secretions, with the various disorders due to the arrest of these, and of the exhalations, as, also, the molecular changes of nutrition, disturbance of colorification, &c., are, in like manner, attributable to the nervous system, although the disorder of the latter has been induced by an ulterior influence exerted on the fluids by the primary, direct action of the remote cause.—Does not this view appear to reconcile the two doctrines in the explanation of the phenomena of fever, as regards their sources and order of catenation?

So far, however, we have considered the first link in the chain as existing in the fluids. But are all fevers of this humoral origin? If this question can be answered negatively, it will render the reconcilement of the two doctrines more complete. We have said that in the instances of fevers from animal poison or malaria, it seems most rational to think that the fluids are primarily affected, although the characteristic phenomena of the febrile state, may, perhaps, be attributed to the nervous system, which becomes, as may be supposed, secondarily affected. Now the inquiry arises: is it not probable that the same modification of the nervous system may ensue from the operation of causes which exert an immediate effect upon this system, not involving a preceding change in the fluids? It is by no means difficult to conceive of the affirmative of this inquiry being true. In this way powerful impressions made directly on the nervous system through the mind, through the senses, and by means of impressions not developed to the perceptions of the individual, nor appreciable in their character and agency, we can readily believe, may create fever, by inducing a morbid condition of this system, which occasions that temporary derangement of the whole economy characteristic of the febrile career. At the same time, unbiased reflection on the subject, will, probably, lead to the conviction that most fevers, and especially epidemic, endemic, and contagious fevers, involve, as the first change, perver-[illegible] the fluids.

The foregoing views appear to the mind of the writer best to comport with the present state of our knowledge respecting thegeneral pathology of Fever. They are submitted for the consideration of those who have a fondness for speculative reflections of this sort. They have, at least, the advantage of in some measure harmonizing doctrines, which, when exclusively maintained, are separated by a wide space from each other. While we are free to admit that the aphorism, in mediis tutissimus ibis, possesses no intrinsic virtue, either as an argument, or as a rule of conduct for the understanding in its progress after truth, it must also be admitted, that with the present relations of medical opinions, the safest policy both for the theorist and the practitioner, is that of a rational eclecticism. *October*, 1845.

